# The Emerging Role of the Serine Incorporator Protein Family in Regulating Viral Infection

**DOI:** 10.3389/fcell.2022.856468

**Published:** 2022-04-01

**Authors:** Shaofen Xu, Zhichao Zheng, Janak L. Pathak, Haoyu Cheng, Ziliang Zhou, Yanping Chen, Qiuyu Wu, Lijing Wang, Mingtao Zeng, Lihong Wu

**Affiliations:** ^1^ Guangzhou Key Laboratory of Basic and Applied Research of Oral Regenerative Medicine, Guangdong Engineering Research Center of Oral Restoration and Reconstruction, Affiliated Stomatology Hospital of Guangzhou Medical University, Guangzhou, China; ^2^ Department of Basic Oral Medicine, Guangzhou Medical University School and Hospital of Stomatology, Guangzhou, China; ^3^ Center of Emphasis in Infectious Diseases, Department of Molecular and Translational Medicine, Paul L. Foster School of Medicine, Texas Tech University Health Sciences Center El Paso, El Paso, TX, United States; ^4^ Vascular Biology Research Institute, Guangdong Pharmaceutical University, Guangzhou Higher Education Mega Center, Guangzhou, China

**Keywords:** SERINC, retroviral virus, DNA virus, HIV, COVID-19, influenza virus

## Abstract

Serine incorporator (SERINC) proteins 1–5 (SERINC1-5) are involved in the progression of several diseases. SERINC2-4 are carrier proteins that incorporate the polar amino acid serine into membranes to facilitate the synthesis of phosphatidylserine and sphingolipids. SERINC genes are also differentially expressed in tumors. Abnormal expression of SERINC proteins occurs in human cancers of the breast, lung, colon, liver, and various glands, as well as in mouse testes. SERINC proteins also affect cleft lip and palate and nerve-related diseases, such as seizure Parkinsonism and borderline personality. Moreover, SERINC proteins have garnered significant interest as retroviral restriction factors, spurring efforts to define their function and elucidate the mechanisms through which they operate when associated with viruses. Human SERINC proteins possess antiviral potential against human immunodeficiency virus (HIV), SARS-COV-2, murine leukemia virus (MLV), equine infectious anemia virus (EIAV), and hepatitis B virus (HBV). Furthermore, the crystal structure is known, and the critical residues of SERINC5 that act against HIV have been identified. In this review, we discuss the most prevalent mechanisms by which SERINC3 and SERINC5 antagonize viruses and focus on the potential therapeutic applications of SERINC5/3 against HIV.

## Introduction

**FIGURE 1:**
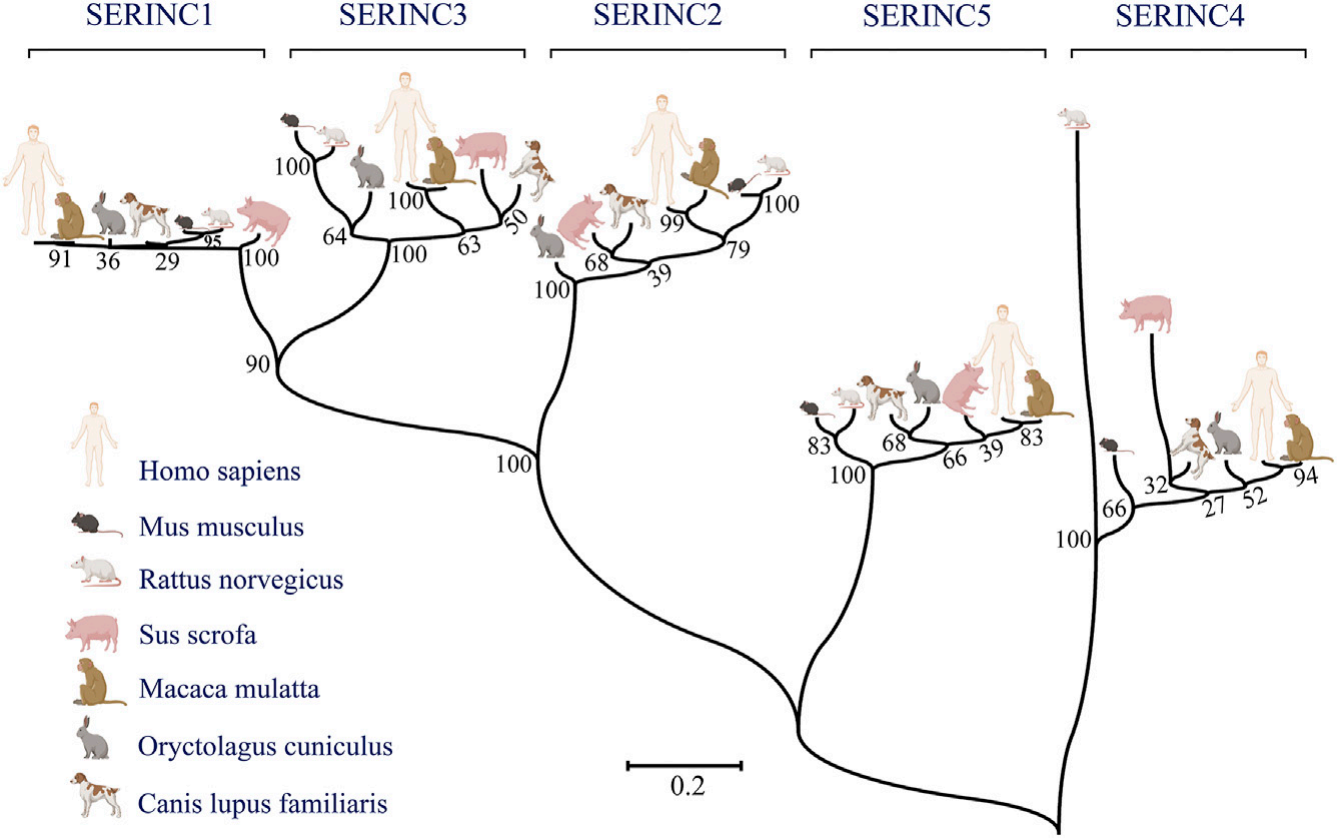
The phylogenetic relationships between humans and other commonly used experimental animals are depicted using MEGA7 software. Maximum likelihood was chosen as the statistical method. The bootstrap value was set to 1,000 gaps/missing data treatment is complete deletion. (Created by Biorender.com).

In mammals, the serine incorporator (SERINC) family of proteins is a multiple-transmembrane-segment family composed of five members, SERINC1-5 (Firrito et al., 2018). SERINC2-4 incorporate serine, a nonessential polar amino acid, into cell membranes and facilitate the synthesis of two serine-derived lipids, phosphatidylserine and sphingolipids (Inuzuka et al., 2005; Chu et al., 2017; Trautz et al., 2017). The topological structure of the SERINC family includes 10–11 transmembrane segments, similar to amino acid transporters (Qiu et al., 2020). Pye et al. used cryo-electron microscopy to determine the structure of human SERINC5 protein expressed in *Drosophila melanogaster*, which revealed a novel fold comprised of ten transmembrane helices organized into two subdomains and bisected by a long diagonal helix (Pye et al., 2020). Furthermore, hydropathy analysis revealed that SERINC family members contain 53%–58% hydrophobic amino acids clustered into 11 regions of up to 30 amino acids in length, suggesting membrane-spanning domains (Inuzuka et al., 2005). All family members encode an N-terminal signal peptide and have about 31%–58% amino acid homology in mammals. A phylogenetic tree was constructed according to the amino acid sequences of different species, including human, rhesus monkey, Norway rat, house mouse, pig, rabbit, and dog (Figure 1). Furthermore, SERINC proteins were thought to have highly conserved sequences and no amino acid homology with other proteins (Grossman et al., 2000; Ren et al., 2014). However, in a recent report, Alli-Balogun et al. identified Ice2p as a full-length homolog of SERINC proteins (Alli-Balogun and Levine, 2021).

**FIGURE 2:**
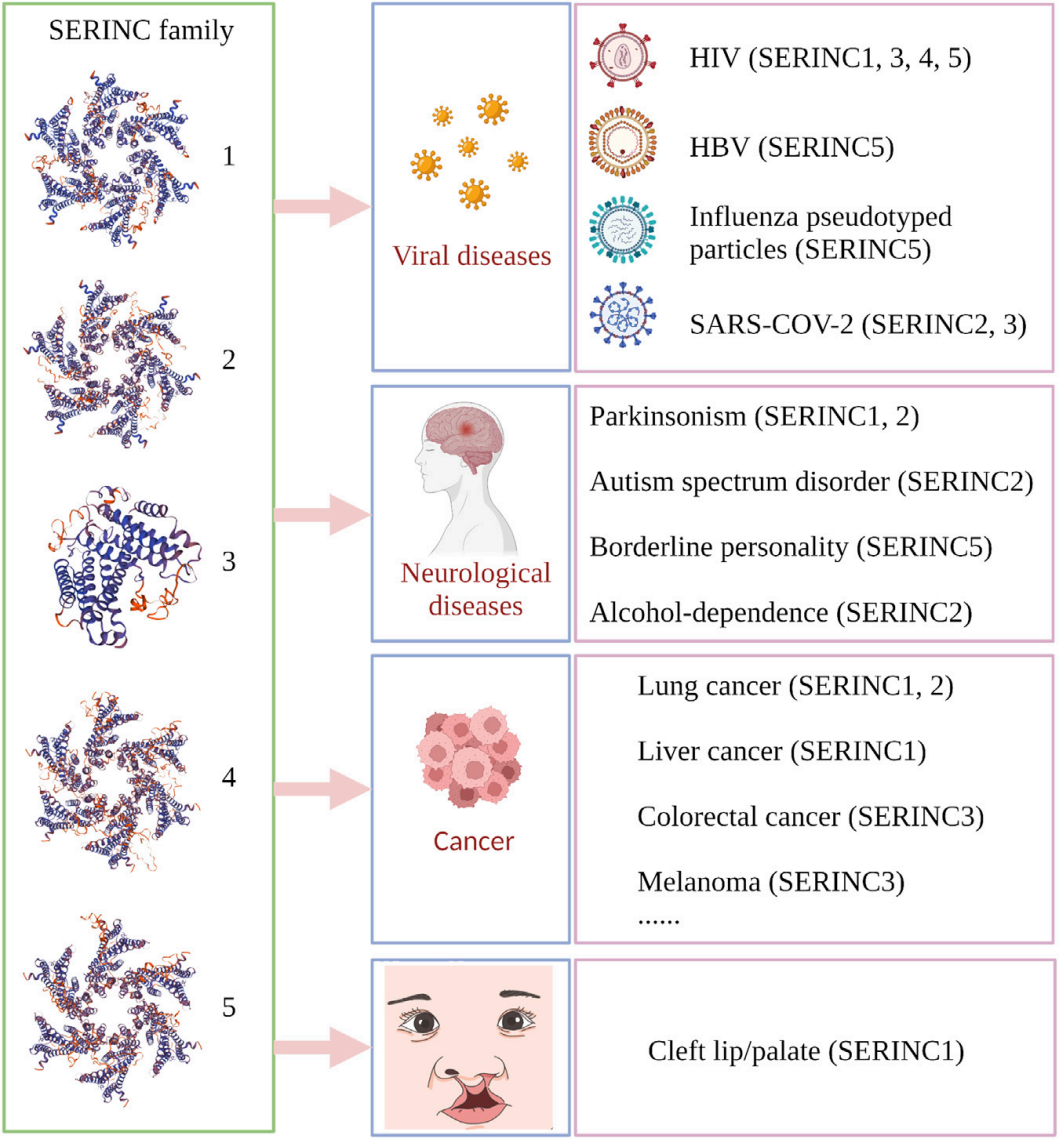
Summary of SERINC-related diseases in human and animal models. Virus-related diseases include acquired immunodeficiency syndrome (HIV; SERINC1, 3, 4, 5), influenza-pseudotyped particles (SERINC5), COVID-19 (SERINC2, 3), and viral hepatitis type B (HBV) (SERINC5). Nerve-related diseases include seizure Parkinsonism (SERINC1, 2), autism spectrum disorder (SERINC2), borderline personality (SERINC5), and alcohol dependence (SERINC2). Cancer types include lung cancer (non-small cell lung cancer and lung adenocarcinoma; SERINC1, 2), liver cancer (SERINC1), colorectal tumor (SERINC3), and melanoma (SERINC3), among others. Animal models include rabies virus (RABV), testicular tumor, and nonobese diabetes. Cleft lip/palate is associated with SERINC1. (Created by Biorender.com).

SERINC family proteins participate in the development of many diseases, such as cancer, nerve-related disease, and other diseases. SERINC1 is associated with the development of lung cancer and hepatocarcinoma (Player et al., 2003; Ren et al., 2014), while SERINC2 is related to the progression of lung adenocarcinoma, low-grade glioma, and leukemia (Zeng et al., 2018; Fang et al., 2020; Qi et al., 2020), and SERINC3 is highly expressed in testicular tumors of polyomavirus large-T antigen transgenic mice (Bossolasco et al., 1999). Moreover, SERINC3 influences the development of lung tumors and colorectal tumors (Bossolasco et al., 1999; Nimmrich et al., 2000). Several reports have shown that the exceptional expression of SERINC proteins in the brain is related to psychiatric disorders, including seizure Parkinsonism, borderline personality, autism-spectrum disorder, and alcohol dependence (Inuzuka et al., 2005; Yeo et al., 2013; Zuo et al., 2013; Lubke et al., 2014; Hnoonual et al., 2017). The disease-regulation mechanisms of the SERINC family are summarized in Figure 2.

According to the International Mouse Phenotyping Consortium (IMPC) database, SERINC2-knockout mice show impaired glucose tolerance (IGT). IGT is an indicator of pre-diabetes, which eventually turns into diabetes, and both conditions predispose patients to cardiovascular alterations. IGT is also associated with increased cardiovascular risk (Khan et al., 2019). Thus, SERINC2 may protect mice from diabetic and cardiovascular-related diseases. SERINC3-knockout mice show a phenotype that includes a decreased circulating magnesium level, decreased bone mineral density, decreased bone mineral content, increased circulating glycerol level, and abnormal auditory brainstem (Beekman et al., 2020). Glycerol is produced by white adipose tissue to process excess glucose, and high levels of circulating glycerin is a known biomarker for increased risk of hyperglycemia and type 2 diabetes (Beekman et al., 2020). Auditory brainstem response (ABR) is a scalp-recorded response to activation of nerve fibers in the auditory nerve and brainstem. The diseases affecting abnormal ABR are auditory neuropathy and vestibular schwannoma, Gaucher disease, Krabbe disease, Bell’s palsy, Duane retraction syndrome, Marcus Gunn ptosis, and various encephalomyopathies (Eggermont, 2019). Thus, SERINC3 may be related to the development of several diseases, such as diabetes, cardiovascular disease, and auditory-related disease. However, the phenotypes of SERINC1- and SERINC4-knockout mice have not yet been tested. Table 1 summarizes the role of SERINC proteins in mouse physiology and metabolism.

**TABLE 1:** The role of the SERINC proteins in mouse physiology and metabolism.

System	SERINC2	SERINC3	SERINC5
Mortality/aging	ns	ns	ns
Embryo	-	-	-
Reproductive system	-	ns	-
Growth/size/body region	ns	ns	ns
Homeostasis/metabolism or adipose tissue	Impaired glucose tolerance**	Decreased circulating magnesium level and increased circulating glycerol level**	ns
Behavior/neurological or nervous system	ns	ns	ns
Cardiovascular system	ns	ns	ns
Respiratory system	-	-	-
Digestive/alimentary or liver/biliary system	-	ns	-
Renal/urinary system	-	-	-
Limbs/digits/tail	-	ns	-
Skeleton	ns	Decreased bone mineral content and density**	-
Immune system or hematopoietic system	-	ns	-
Muscle	-	-	-
Integument or pigmentation	-	ns	
Craniofacial	-	ns	-
Hearing/vestibular/ear	ns	Increased circulating glycerol level**	ns
Taste/olfaction	-	-	-
Endocrine/exocrine gland	-	-	-
Vision/eye	-	ns	ns

Data are from the IPMC (https://www.mousephenotype.org/). Phenotyping is currently not planned for SERINC1- and SERINC4-knockout strains. “-”, not tested; ns, not significant; **p-value <0.001.

Previous studies demonstrated that SERINC proteins are involved in several processes, such as defense against RNA and DNA viruses. It has been shown that SERINC3 and SERINC5 inhibit human immunodeficiency virus (HIV) infection at an early stage of the viral cycle, regulate viral fusion, and act as restriction factors (Rosa et al., 2015; Usami et al., 2015). Bibert et al. reported that SERINC2 is one of the most highly overexpressed genes when comparing transcriptional profiles between severe SARS-COV-2 presentations, infections with influenza A or B, and healthy individuals (Bibert et al., 2021). According to an analysis of expression of quantitative trait loci (eQTLs) of 60 virus restriction factors, genetic control of the expression of SERINC3 may underlie inter-individual differences in risk or severity of infection with SARS-COV-2 (Cotroneo et al., 2021). Here, we review the current knowledge of the entire SERINC family concerning the regulation of viral infections and discuss the possibilities of developing SERINC protein-targeted diagnostic and therapeutic approaches.

## The Interaction Between Viruses and Host Cells

It has been demonstrated that complex interactions between virus and host are involved at each stage of the virus life cycle. Host restriction factors are an integral part of the host defense against a viral pathogen by limiting the virus at each stage of viral replication and sending out continuous pathogen-invasion signals. Host restriction factors and viral accessory proteins are involved in the interaction between viruses and host cells. Viruses seek to penetrate the resistance of host cells, while the host cells seek to prevent entry by the virus invader through complex mechanisms (Konig and Stertz, 2015). Several coding genes and noncoding genes participate in the balance between virus attachment and the defense of host cells. The general processes of virus infection mainly include virus binding (Figure 3①), release of the virus core into the cytoplasm, reverse transcription of the virus genome (Figure 3④), viral DNA integration into the host chromosome (Figure 3⑤), viral protein synthesis and assembly (Figure 3⑥), and release of progeny virus particles (Figure 3⑦) (Fanales-Belasio et al., 2010; Liu et al., 2020). Given their limited genome size, viruses cannot encode all of the proteins required for these processes. Therefore, viruses have evolved mechanisms to hijack and subvert the host cell to achieve these goals (Ramage and Cherry, 2015).

**FIGURE 3:**
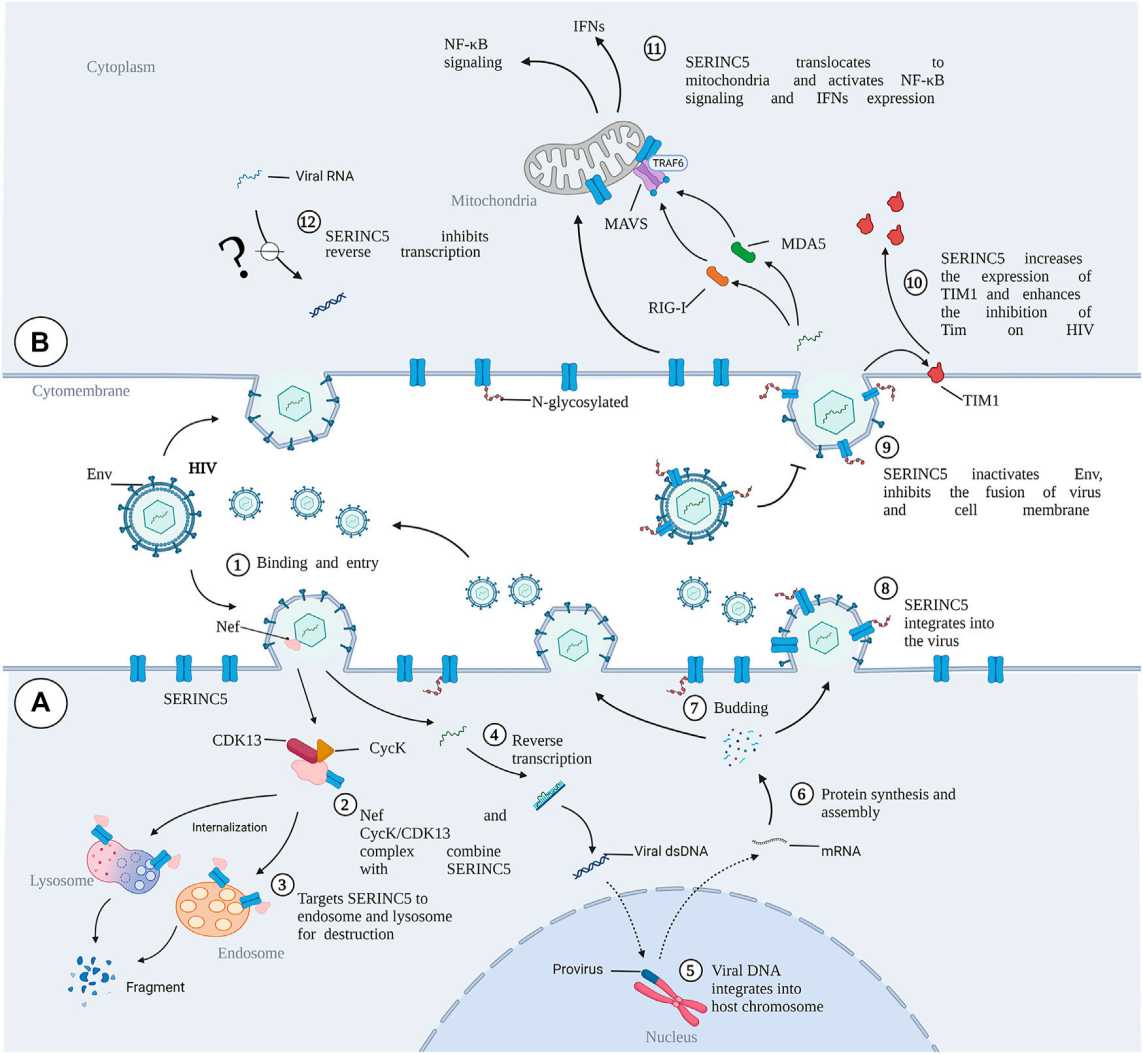
The role of SERINC5 in HIV infection. **(A)** HIV infects cells and antagonizes SERINC5. **(B)** SERINC5 inhibits viral infection. SERINC5 proteins are normally localized in the plasma membrane of the cell. Upon viral infection, Nef forms complexes with CycK and CDK13, combining with SERINC5 to target SERINC5 to endosomes and lysosomes for destruction. During HIV budding, an N-glycosylated and high-molecular-weight form of SERINC5 preferentially integrates into virions. If viruses carrying SERINC5 infect other cells, the packaged SERINC5 will exert several antiviral activities. SERINC5 can interfere with Env activity and prevent Env folding, reducing the fusion between cells. SERINC5 increases the expression of TIM1 and enhances the inhibition of Tim on HIV. SERINC5 becomes internalized and translocates to the mitochondrial membrane, where it is associated with MAVS and TRAF6, resulting in MAVS aggregation and polyubiquitylation of TRAF6. MVAS recognizes viral MDA5 and RIG-I, enhancing the expression of genes encoding type I interferons (IFNs) and nuclear factor κB (NF-κB) signaling. MDA5, melanoma differentiation gene 5; RIG-I, retinoic acid-inducible gene I; Cyck, cyclin K; CDK13, cyclin-dependent kinase 13. (Created by Biorender.com).

Viral accessory proteins are not necessary for replication (Liu et al., 2014). However, they are essential for viral infection and counteracting the responses of host restriction factors (Zheng et al., 2012). The arms race between hosts and viruses is ancient, but viruses can evolve faster than their hosts so that the innate immune system of modern-day vertebrates cannot cope with newer viral threats (Duggal and Emerman, 2012).

Furthermore, restriction factors are crucial for host intrinsic and innate immunity to counter viral invasion and replication. This set of proteins includes constitutively expressed proteins and those induced by interferons (IFNs) (Lerolle et al., 2021). The canonical restriction factors include catalytic polypeptide 3G (APOBEC3), APOBEC3F, bone marrow stromal cell antigen 2 (BST-2), tripartite motif-containing 5α (TRIM5α), and sterile alpha motif and HD-domain-containing protein 1 (SAMHD1), which are stimulated by IFNs and produced by cells after viral infection (Ghimire et al., 2018). The SERINC protein family is a class of identified restriction factors and was first shown to be antiviral in 2015. These proteins target fusing membranes and impair the fusion of viral and host membranes (Rosa et al., 2015; Usami et al., 2015; Chen et al., 2020; Sarute et al., 2021; Sauter and Kirchhoff, 2021). It was found that SERINC5 targets HIV, simian immunodeficiency virus (SIV), and murine leukemia virus (MLV) by acting against the convergent antagonistic retroviral factors Nef and glycoGag, while SERINC3 may also inhibit HIV-1 infectivity (Rosa et al., 2015; Usami et al., 2015). Conversely, accessory factors have an inhibitory effect against SERINC proteins. Unlike typical restriction factors, SERINC proteins are not induced by interferon and are not under strong positive selection (Murrell et al., 2016). In contrast to classical antiviral restriction factors, tumor necrosis factor-alpha (TNF-α), IFNs, and pro-inflammatory interleukins do not affect SERINC protein levels, while upregulating classical HIV innate immunity factors, such as tetherin (BST2 and CD317) or interferon γ-inducible protein 16 (IFI16) (Zutz et al., 2020). Furthermore, SERINC proteins are not dysregulated in CD4^+^ T cells and monocytes isolated from chronic HIV-1-infected patients (Zutz et al., 2020). SERINC5 is upregulated during myeloid cell differentiation, which would be expected for an innate immunity factor (Zutz et al., 2020). Interestingly, a new study demonstrated that SERINC3 and SERINC5 promote innate immune signaling, resulting in increased production of type I IFNs and pro-inflammatory cytokines, thereby inhibiting the infection of HIV-1, vesicular stomatitis virus (VSV), and Zika virus (ZIKV). After infection of cells with Sendai virus or treatment with poly (I:C), SERINC5 is recruited to mitochondria, where it is colocalized and interacts with mitochondrial antiviral signaling protein (MAVS), enhancing its polymerization. SERINC5 also interacts with and stabilizes a tumor necrosis factor receptor-associated factor (TRAF6), suggesting a model in which SERINC5, MAVS, and TRAF6 form a signaling complex in mitochondria. This work showed that SERINC5 is a positive regulator of type I IFN expression (Figure 3 ⑪) (Zeng et al., 2021).

## SERINC Proteins and Antagonistic Retroviral Factors

There are several types of SERINC family members that mainly counteract primate lentivirus (simian immunodeficiency virus, SIV), gammaretrovirus (MLV), equine infectious anemia virus (EIAV), and hepatitis B virus (HBV) viral protein (Rosa et al., 2015; Chande et al., 2016; Kmiec et al., 2018; Liu et al., 2020). SERINC5, SERINC3, and SERINC1 are essential elements in antagonizing retroviral factors (Goffinet, 2016). Recently, it was found that the significantly increased expression of SERINC4 has a strong inhibitory effect against HIV (Qiu et al., 2020). Matheson et al. found Nef to be sufficient for downregulation of the serine carriers SERINC3 and SERINC5 but increases the surface abundance of SERINC1 (Matheson et al., 2015). Schulte et al. demonstrated that human SERINC2 does not restrict viruses or integrate into viral particles effectively (Schulte et al., 2018). Therefore, SERINC2 is suitable for constructing a chimera to study the restriction factor determinant region. These experiments using SERINC5–SERINC2 chimeric proteins revealed two functional domains essential for SERINC2 incorporation into viral particles and changing the HIV-1 envelope conformation (Schulte et al., 2018). Ramdas et al. confirmed that SERINC2 in coelacanths limits HIV-1 infection. However, the antiviral effect of SERINC2 is lost with evolution. This loss in human SERINC2 is associated with its post-whole-genome duplication (post-WGD) divergence (Ramdas et al., 2021). However, HIV Nef and MLV glycoGag do not restrict coelacanth SERINC2. Foamy virus (FV) envelope protein enhances virus infectivity by preventing virus particles containing SERINC2 from merging (Schulte et al., 2018). Thus, SERINC2 may constitute a key barrier against FV in the early stages, and the loss of activity of SERINC2 homologous protein may be related to this new function (Ramdas et al., 2021). SERINC3 and SERINC5 are involved in regulating HIV, MLV, EIAV, and other viruses (de Sousa-Pereira et al., 2019).

### SERINC Proteins and RNA Viruses

#### SERINC Proteins and HIV-1

HIV, a typical RNA retrovirus, includes two lentiviruses, HIV-1 and HIV-2, and HIV-1 is the main causative factor of acquired immunodeficiency syndrome (Sharp and Hahn, 2011). Reports demonstrated that the ability of progeny HIV-1 virions to fuse with target cells is abolished by SERINC5, thus strongly inhibiting the ability of HIV-1 virions to enter target cells (Rosa et al., 2015; Usami et al., 2015). Assisting the development of animal models of HIV-1, the antiviral activity of SERINC3/5 protein was found to be conserved in rodents and lagomorphs and is overcome by HIV, MLV, and EIAV. The SERINC3/5 gene homolog in rodents and lagomorphs, therefore, does not pose any obstacle to the development of an HIV-1 animal model (de Sousa-Pereira et al., 2019). Nef is a myristoylated protein that promotes HIV infectivity and is expressed in the early stages of the virus replication cycle. A prominent role of Nef is to downregulate cell-surface molecules by localizing them in the vesicular machinery. Mostly, Nef downregulates CD4 and MHC-I molecules (Pereira and Dasilva, 2016). SERINC5 has several spliced isoforms, and SERINC5-001 has been identified as the predominant isoform for HIV restriction against Nef (Zhang et al., 2017). It has been found that HIV-1 Nef binds with SERINC5 and downregulates it via the endosome/lysosome system (Shi et al., 2018). In the presence of Nef, SERINC5 is downregulated from the cell surface and relocated to Rab5^+^ early, Rab7^+^ late, and Rab11^+^ recycling endosomes. However, SIV Nef promotes SERINC5 degradation via the proteasome pathway (Kmiec et al., 2018). Chai et al. used affinity purification/mass spectrometry to identify a complex of cyclin K (CycK) and cyclin-dependent kinase 13 (CDK13) that interacts with Nef to antagonize SERINC5 for optimal viral infectivity (Figure 3②). Mechanistically, the CycK–CDK13 complex phosphorylates the serine at position 360 in SERINC5, resulting in downregulation of SERINC5 from the cell surface (Chai et al., 2021) (Figure 3③). With the help of Nef, HIV and SIV have evolved to escape immune responses, especially from CD8^+^ T lymphocytes (Schouest et al., 2018). Schoues et al. further demonstrated that the variation of each residue within the range of positions 195–203 in Nef could affect anti-SERINC activity, which is driven by CD8TL (Schouest et al., 2018). Naturally occurring mutations in Nef also affect its ability to resist the inhibition of viral infection by SERINC3 and SERINC5, thus influencing the viral load in plasma (Toyoda et al., 2020; Kruize et al., 2021). Similarly, the antagonistic activity of SERINC3 and SERINC5 varies markedly among circulating Nef isolates and between viral subtypes, indicating that variation in Nef-mediated SERINC antagonism may lead to differences in pathogenesis among HIV subtypes (Jin et al., 2020).

With a molecular weight of 55 kDa and modification by N-linked complex glycans, SERINC5 is preferentially integrated into virions (Figure 3⑧) (Sharma et al., 2018). Although N-linked glycosylation is not necessary for SERINC5 activity or Nef sensitivity, it is important for maintaining the stable expression of SERINC5. Non-glycosylated SERINC5 may be degraded in proteasomes. It has been demonstrated that the sensitivity of SERINC5 against HIV is different in vertebrates (Dai et al., 2018). The 10th transmembrane domain of SERINC proteins is important for self-stabilization and HIV suppression (Zhang et al., 2017), while the long loop between domains 7 and 8 determines the sensitivity of SERINC to Nef (Dai et al., 2018). Human SERINC5 shows Nef sensitivity, while the SERINC5 in frog is Nef resistant (Dai et al., 2018). A long cytoplasmic loop governs the sensitivity of SERINC5 against HIV-1 Nef. When the intracellular loop 4 (ICL4) of Nef-resistant SERINC5 in frog is replaced by Nef-sensitive human SERINC5, Nef-resistant SERINC5 becomes sensitive to Nef. By contrast, when human ICL4 is replaced by Nef-resistant SERINC5, human SERINC5 becomes resistant to Nef (Dai et al., 2018). Mechanistically, a leucine residue (L350) among residues 9–26 of ICL4 in human SERINC5 is responsible for its sensitivity to Nef (Dai et al., 2018) (24), while deletion of the conserved amino acid sequence “EDTEE”, which is located within a cytoplasmic loop of SERINC5, increases the antagonism of SERINC5 by Nef (Stoneham et al., 2020). Moreover, the presence of a phosphoserine acidic cluster motif contained in the amino acid sequence “SGASDEED” in the cytoplasmic loop (loop 10) of SERINC3 may have a membrane-sorting or trafficking function and have no impact on the sensitivity to Nef (Stoneham et al., 2020). The aromatic side chain at position 412 of SERINC5 plays a critical role in mediating antiviral functions toward HIV-1 and other retroviruses (Tan et al., 2021).

It has been demonstrated that gp120, the surface subunit of the envelope glycoproteins (Env) in HIV-1, recognizes target cells, while gp41, the transmembrane subunit of Env, mediates the membrane fusion of HIV-host cells and the cytoplasmic release of the virus core (Weiss, 2003; Chen, 2019). In the presence of SERINC5, the content of Env protein in HIV is decreased. However, the Env structure and glycosylation level are not changed (Chen et al., 2020). SERINC5 reduces HIV-1 capture of the specific gp120-epitope antibodies, which indicates that SERINC5 can alter the conformation of gp120 (Featherstone and Aiken, 2020). It has been demonstrated that SERINC5 inhibits HIV and host cell fusion during pore formation, which results from spontaneous inactivation of sensitive Env, changes of gp41 natural trimer structure, and the delay of gp41 refolding into its final trimer hairpin structure (Sood et al., 2017). SERINC3 and SERINC5 reduce fusion between the Env proteins of offspring virus and host cells, ultimately limiting the ability of the offspring virus to infect new target cells (Figure 3⑨). However, Env proteins of certain HIV-1 strains have evolved to overcome SERINC5 inhibition to efficiently infect host cells (Rosa et al., 2015). Five variable regions have been designated in gp120 (V1–V5). The V1/V2 domain is involved in viral tropism during infection, and the V3 loop is one of the primary determinants of viral tropism (Hoffman and Doms, 1999; Granados-Gonzalez et al., 2009). Env V1 and V2 loops have been shown to allow Env to counter SERINC5 (Usami and Gottlinger, 2013; Usami et al., 2015). Beitari et al. confirmed that the Env V3 loop plays a role in antagonizing SERINC5. Because the V1 and V2 loops fold into a pocket where the V3 loop resides, the authors speculated that V1, V2, and V3 are interdependent structural entities modulating the stability of Env protein, which function together to resist SERINC5 (Beitari et al., 2017). In addition, the Env cytoplasmic tail (EnvCT) mediates both intensive Env intracellular trafficking and interactions with numerous cellular and viral proteins, optimizing viral infectivity (Da Silva et al., 2013). Haider et al. further showed that EnvCT truncation changes the conformation and function of Env, thus disarming the restriction ability of SERINC5 (Haider et al., 2021). Furthermore, HIV-1 Env conformation changes and CD4 expression on the surfaces of target cells are essential for SERINC5-mediated viral infectivity (Zhang et al., 2019; Featherstone and Aiken, 2020; Staropoli et al., 2020; Diehl et al., 2021). T-cell immunoglobulin and mucin domain (TIM), a host restriction factor, regulates the host immune response by binding with phosphatidylserine (PS). Nef upregulates TIM-3 cell-surface levels of the checkpoint protein, internalizes TIM-1 from the plasma membrane, and isolates TIM-1 in the intercellular septum (Evans and Liu, 2020; Jacob et al., 2021). Li et al. further found that SERINC proteins may enhance TIM-mediated inhibition of HIV-1 release by stabilizing TIM-1 expression (Figure 3⑩) (Li M. et al., 2019).

To further reveal antiviral activity, Pye et al. generated the three-dimensional structures of human SERINC5 and its ortholog from *Drosophila melanogaster* at subnanometer and near-atomic resolutions, respectively (Pye et al., 2020). Some critical and conserved residues, such as K130, F165, and Y388, have been identified for Nef-defective HIV-1_NL4-3_ restriction and surface expression of SERINC5 (Pye et al., 2020). These results demonstrated the importance of resistance to HIV-1 Env protein by SERINC5. The regulatory mechanism is shown in Figure 3.

#### SERINC Proteins and H7/N1-Pseudotyped HIV-1 Particles

Influenza virus and HIV-1 display type I fusion proteins, and influenza virus can be divided into several subtypes according to hemagglutinin (HA) and neuraminidase (NA) serotypes. Recently, it was reported that SERINC5 has inhibitory activity against H7/N1-pseudotyped HIV-1 particles (Diehl et al., 2021), although other proteins in the SERINC family have no inhibitory function against the virus. A possible reason is that SERINC5 inhibits the infectivity of HA/NA pseudovirus to target cells by incorporating them into pseudovirus particles. Moreover, the inhibitory activity of SERINC5 against the infectivity of HA/NA pseudovirus particles is inhibited by wild-type Nef, while other cofactors of influenza virus do not affect SERINC5 activity (Diehl et al., 2021). However, an inhibitory effect of SERINC proteins on influenza virus has not been reported.

#### SERINC Proteins and SARS-COV-2

SARS-COV-2 belongs to the Coronaviridae family, *Betacoronavirus* genus, and subgenus *Sarbecovirus* (Ciotti et al., 2019) and has rapidly spread all over the world, causing the COVID-19 pandemic. By June 2021 it had caused almost four million deaths (Guner et al., 2021). Bibert et al. compared the transcriptional profiles of SARS-COV-2-infected patients with influenza A or B patients having different levels of disease severity and healthy individuals. The stratification of SARS-COV-2 patients included groups that did not require oxygen support (OXY0), that received oxygen but no mechanical ventilation (OXY1), or that required mechanical ventilation (TUBE), according to the level of respiratory failure. SERINC2 is significantly increased in early (TUBE)-treated compared with OXY0- or OXY1-treated SARS-COV-2 patients, influenza A or B-infected patients, or healthy individuals. Moreover, early TUBE patients represent sampling within 7 days of hospitalization (Bibert et al., 2021). Prokop et al. detected 770 genes whose transcript levels are significantly altered in the blood of SARS-COV-2 patients (Prokop et al., 2021). SERINC2 is highly expressed in patients and has a correlation of 0.4–0.5 with the simplified acute physiology score II (SAPSII), which is a standard intensive care metric that integrates multiple clinical annotations to predict disease severity and mortality risk (Prokop et al., 2021). Cis expression quantitative trait loci (Cis-eQTLs) of SERINC3 were identified as putative SARS-COV-2 infection-associated genes in lung tissue (Cotroneo et al., 2021). These results support a role for these loci in susceptibility to severe COVID-19. Lung samples from SARS-COV-2 patients and healthy persons were not used. Therefore, the function of SERINC3 in COVID-19 remains to be investigated.

#### SERINC Proteins and Other RNA Viruses

MLV is a gammaretrovirus, and glycosylated Gag (glycoGag) is an accessory protein expressed by MLV, which reduces the expression of SERINC5 protein in mice through the endosomal/lysosomal pathway. Two key residues, P31 and R63, of MLV glycoGag are important for the regulation of SERINC5. The interaction between glycoGag and SERINC5 in living cells leads to the relocation of SERINC5 from the plasma membrane to the spotted compartment around the nucleus, and the Y36XXL39 motif of MLV glycoGag is essential in this process (Li S. et al., 2019). EIAV S2 protein can replace the activity of HIV-1 Nef and antagonize the SERINC protein. Mechanistically, EIAV S2 protein recruits AP-2, relocates SERINC3 and -5 to late endosomal vesicles and promotes SERINC5 degradation, thus preventing the integration of SERINC3 and SERINC5 into offspring virus particles. It has been demonstrated that Env also regulates the susceptibility of EIAV to SERINC5 (Chande et al., 2016). Timilsina et al. first showed the potent antiviral effect of SERINC5 on MLV with both glycoGag and viral Env in transgenic mice, while SERINC3 has no antiviral effect (Timilsina et al., 2020). Furthermore, the growth of classical swine fever virus (CSFV) is significantly inhibited by SERINC5 overexpression in PK-15 and 3D4/2 cells, while SERINC5 silencing enhances the proliferation of CSFV. Further studies showed that SERINC5 inhibits CSFV replication by activating the melanoma differentiation-associated gene 5 (MDA5)-mediated type I IFN signaling pathway (Li et al., 2020). Changes in the circular RNA spectrum were detected in the brains of Rabies virus (RABV)-infected or uninfected mice by RNA sequencing, and RABV infection was found to significantly change the expression pattern of 636 circular RNAs. Among these circular RNAs, SERINC3 significantly improved novel_circ_017745 expression, while the mRNA expression pattern of host genes remained unchanged with RABV infection (Zhao et al., 2021).

Some non-retroviruses may be sensitive to SERINC5. It has been demonstrated that SERINC5 inhibits glycoprotein pseudoforms of several retroviruses, including HIV-1, A-MLV, RD114, M-MPV, orthomyxovirus (influenza A virus, IAV), rhabdovirus (RABV), paramyxovirus (parainfluenza virus 5, PIV5), and sand virus (lymphocytic choriomeningitis virus, LCMV). The retroviral core also affects the sensitivity of the virus to SERINC5 (Diehl et al., 2021).

## SERINC Proteins and DNA Viruses

HBV, an enveloped and hepatophilic DNA virus, antagonizes SERINC3 and SERINC5 through large (LHB), mid-sized (MHB), and small (SHB) surface proteins of HBV. By contrast, SERINC3 binds to LHBs, relocalizes these proteins to the Golgi apparatus, and reduces the glycosylation modification of envelope proteins, inhibiting HBV secretion. Transmembrane domains 4–6 of SERINC proteins are important for glycosylation modification and HBV inhibition (Liu et al., 2020).

## Perspectives

As mentioned above, the SERINC protein family is involved in the progression of viral infection. During the process of viral infection, SERINC-family proteins are a constitutive host resistance factor, which inhibits viral infection by integrating into virus particles. Currently, the viruses known to be inhibited by the SERINC family include HIV, SIV, MLV, EIAV, HBV, influenza virus, CSFV, and FV (Rosa et al., 2015; Chande et al., 2016; Kmiec et al., 2018; Li et al., 2020; Liu et al., 2020; Ramdas et al., 2021). Thus, the main aim of the strategies against these virus infections should be to prevent the virus from entering a new host, inhibit the fusion of virus and target cell membrane and the release of viral small molecules, as well as antagonize virus helper protein and inhibit translocation of the envelope protein.

SERINC3 and five are correlated with virus resistance. The antiviral function of the SERINC family has been widely investigated. However, the details of the regulatory mechanism, including the identity of the transcription factor regulating SERINC family transcription and the molecules directly interacting with the SERINC family, should be investigated to reveal its antiviral network. Although cell therapy and gene therapy of some restriction factors against retroviruses have been assessed in clinical trials, the therapeutic potential of the SERINC family against viral diseases has not yet been properly investigated (Bhoj et al., 2016).

**FIGURE 4:**
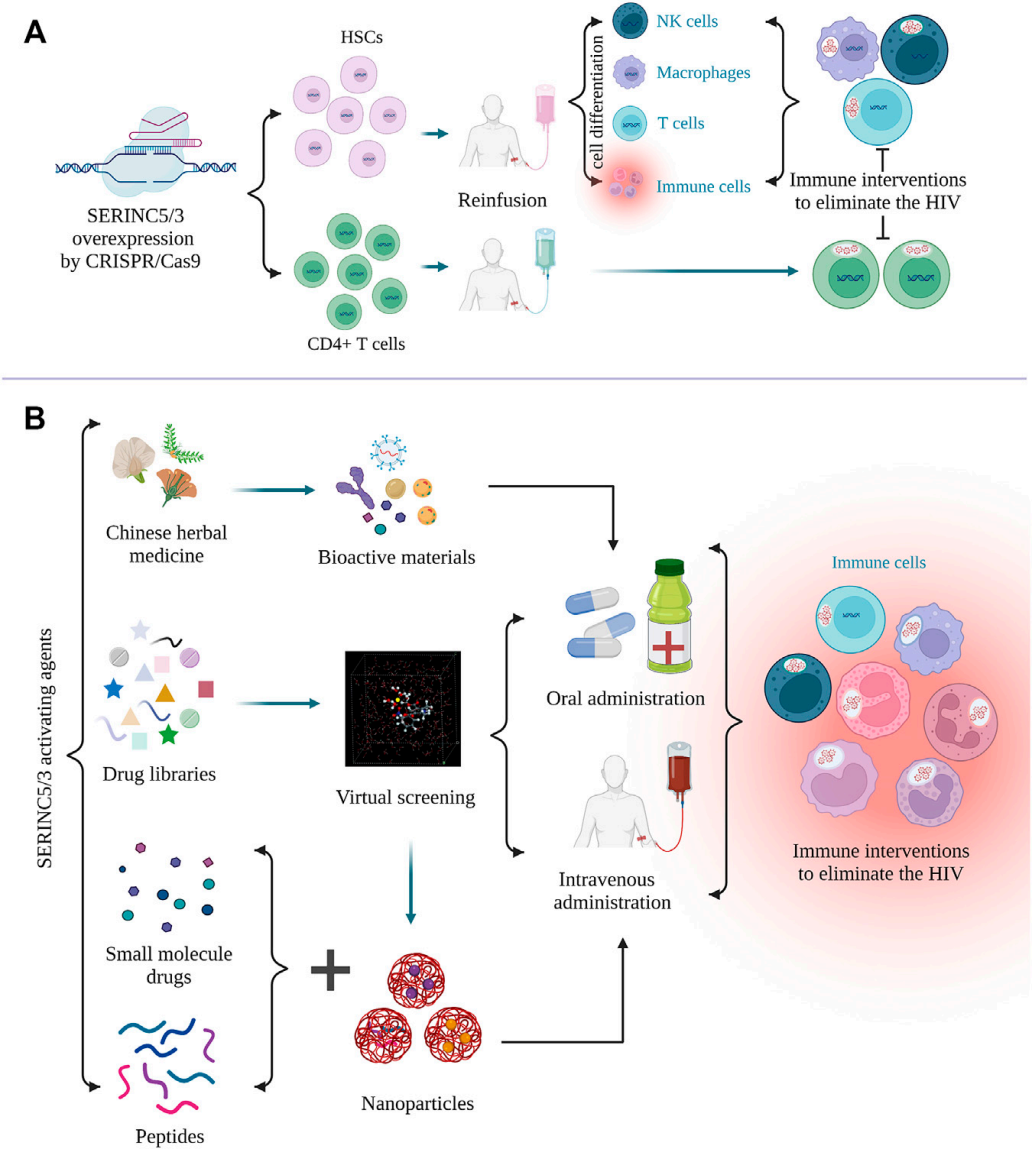
Targeting strategies against HIV by regulating SERINC5/3. **(A)**, CRISPER/Cas9 gene editing can be used to overexpress SERINC5/3 to increase the differentiation of human-induced pluripotent stem cells (iPSCs) into different immune cells, such as T cells and macrophages. SERINC5/3 was overexpressed in CD4^+^ T cells, and these immune cells can eliminate HIV loads. **(B)**, To activate SERINC5/3 modification activity or increase the protein expression of SERINC5/3 to defend against HIV, active materials, such as monomers in Chinese herbal medicine, small molecules, and peptides should be selected and their activity confirmed. Using the crystal structure of virus binding to the SERINCs, additional drugs from molecular libraries can be found by virtual screening. To efficiently deliver the selected drugs or peptides, nanoparticles should be considered as a carrier. (Created by Biorender.com).

C-C motif chemokine receptor 5 (CCR5) is the major cofactor required for HIV entry into the cell. Gene editing of CCR5 on autologous CD4^+^ T cells has proved to be effective and safe in HIV-positive patients (Maier et al., 2013; Tebas et al., 2014). Kang et al. also demonstrated the feasibility and safe production of autologous CCR5-deficient human induced pluripotent stem cells (iPSCs) in HIV-positive patients (Kang et al., 2015). Thus, SERINC5/3 overexpression in CD4^+^ T cells and iPSCs by CRISPR/Cas9 gene editing should also be considered, and the antiviral effects evaluated. SERINC5/3-edited iPSCs may be differentiated into different immune cells, such as macrophages, natural killer cells, and T cells, *in vivo* to reduce viral load. According to the AlphaFold Protein Structure Database (https://alphafold.ebi.ac.uk), screening for activating drugs for SERINC3 and -5 may increase the number of potential targets. Although the crystal structure and critical residues of SERINC5 have been determined (Pye et al., 2020), residue modification of SERINC5, an important factor in protein activity against different kinds of viruses, should be explored (Kao et al., 2004; Song et al., 2020). High-throughput virtual screening could be performed using the crystal structure of SERINC5 or that of the interaction between SERINC proteins with HIV-1. Molecular libraries containing the bio-active agents in Chinese herbal medicine, small molecules, or peptides may contribute to drug development. To our knowledge, no specific activators or drugs targeting SERINC3 or SERINC5 to increase their activity or expression level for antiviral activity have been reported. Furthermore, the drugs against viral accessory proteins that inactivate SERINC proteins should also be explored. To efficiently deliver the selected drugs, engineered materials such as nanoparticles should be optimized to reduce drug degradation and improve target specificity (Oti, 2020). The targeting strategies against HIV are summarized in Figure 4. Based on recent advances in SERINC family protein-related research, development of gene therapy or specific drugs targeting SERINC proteins to treat various diseases is sure to follow.
